# The Association between Clinical Response to Ustekinumab and Immunogenicity to Ustekinumab and Prior Adalimumab

**DOI:** 10.1371/journal.pone.0142930

**Published:** 2015-11-13

**Authors:** Hsien-Yi Chiu, Thomas Waitao Chu, Yu-Pin Cheng, Tsen-Fang Tsai

**Affiliations:** 1 Institute of Biomedical Engineering, College of Medicine and College of Engineering, National Taiwan University, Taipei, Taiwan; 2 Department of Dermatology, National Taiwan University Hospital Hsin-Chu Branch, Hsinchu, Taiwan; 3 Department of Dermatology, National Taiwan University Hospital and National Taiwan University College of Medicine, Taipei, Taiwan; 4 Department of Dermatology, Far Eastern Memorial Hospital, New Taipei, Taiwan; 5 Department of Dermatology, Cathay General Hospital, Taipei, Taiwan; University of Leicester, UNITED KINGDOM

## Abstract

**Background:**

Immunogenicity due to antidrug antibodies (ADA) to tumor necrosis factor (TNF)-α antagonists is known to decrease treatment response. However, few studies have investigated ADA in ustekinumab, an interleukin-12 and -23 antagonist, in a clinical setting. This study aimed to investigate the immunogenicity of ustekinumab and its clinical consequences in psoriasis.

**Methods:**

This prospective observational study enrolled 76 patients with plaque psoriasis who were treated with ustekinumab for a minimum of 7 months. Blood samples were drawn just prior to scheduled ustekinumab injection during clinic visits. Levels of anti-ustekinumab antibody (AUA) and serum ustekinumab concentration were measured respectively by radioimmunoassays and enzyme-linked immunoassays respectively, and correlated to clinical data and Psoriasis Area and Severity Index (PASI).

**Results:**

AUA was detected in 6.5% of patients after a mean of 13 months of treatment. Patients with positive AUA had significantly lower serum ustekinumab concentrations (0.01 vs. 0.2 mg/L, p<0.001) and lower PASI 50 response than patients without AUA (0% vs. 69%, p = 0.004).The percentage of AUA formation was comparable between patients who had failed previous adalimumab with or without anti-adalimumab antibodies (AAA) (14.3% vs. 12.5%, p = 1.00). However, a higher proportion of switchers without AAA obtaining PASI50 (71.4% vs. 37.5%) and PASI75 response (42.9% vs.12.5%) within 7 months of ustekinumab treatment than with AAA though this difference did not reach statistical significance.

**Conclusions:**

Our results suggest that presence of AUA was significantly associated with treatment failure for ustekinumab, though limited by a small sample size. Also, determining the presence of ADA to antecedent TNF-α antagonists may assist in choosing an optimized subsequent treatment modality achieving treatment success.

## Introduction

Psoriasis is as an inflammatory disorder involving increased production of proinflammatory cytokines by the immune system [1,2]. Biologics targeting tumor necrosis factor (TNF) or interleukins 12 and 23 (IL-12/23) are increasingly used to treat moderate-to-severe psoriasis [3,4,5]. Though the majority of patients respond well, a gradual decrease in efficacy over time following an initial response to biologics is common [5]. The presence of antidrug antibodies (ADA) to TNF-α blockers is thought to play a role in secondary treatment failure in patients with rheumatoid arthritis, Crohn’s disease and ankylosing spondylitis [5,6,7,8]. In psoriasis patients treated with TNF-α blockers, a recent systemic review [9] as well our study [10] also suggested that presence of ADAs to infliximab and adalimumab is associated with loss of treatment response [9].

Ustekinumab, a human monoclonal antibody against the shared p40 subunit of IL-12 and IL-23, has shown great benefit in the treatment of psoriasis across different ethnic groups and geographical regions [11,12,13,14,15,16,17]. However, there is a paucity of data on the immunogenicity of ustekinumab in psoriasis patients, particularly for Asians in clinical setting. Much of the existing data—based mostly on Caucasian patients—have not established an association between anti-ustekinumab antibody (AUA) and clinical response. There is also a lack of transparency in various assays used to measure ADA and serum drug concentration, and methods in some clinical trials are not completely reported. It has also been reported that ADAs in a real world may develop at a higher frequency than those reported in clinical trials [9,10,18,19]. To bridge this gap of knowledge, we investigated the risk of ADA formation against ustekinumab in a real world clinical setting and assessed its effect on therapeutic response in a Taiwanese population with psoriasis. Moreover, as a sub-aim of this study, we also evaluated whether formation of ADA to an antecedent biologics, i.e., adalimumab, was associated with lack of clinical response to subsequent ustekinumab treatment.

## Materials and Methods

### Study population

This prospective observational cohort study enrolled 76 consecutive patients with plaque psoriasis who underwent an ustekinumab treatment regimen for at least 7 months at a tertiary referral center between March 2012 and December 2014. The study was approved by the local investigational research bureau of National Taiwan University Hospital (201207080RIC) and National Taiwan University Hospital Hsin-Chu Branch (103-082-E). Patient records/information was anonymized and de-identified prior to analysis. After approval by institutional ethics committee and written informed consent, blood samples were obtained during routine clinic visits for the measurement of AUA and serum ustekinumab concentration. Most psoriasis patients received subcutaneous ustekinumab 45 mg at weeks 0, 4, then every 12 weeks thereafter. Dose reduction was only noted in 12 non-reimbursed patients. In Taiwan, patients with Psoriasis Area and Severity Index (PASI) ≥ 10 who failed conventional systemic agents and phototherapy are eligible for biologics reimbursed by the National Health Insurance. Reimbursement is discontinued for patients with PASI < 10 after 6 months of biologics therapy, and a minimum PASI50 response is required for reapplication. Prior to AUA measurement, the following clinical parameters were recorded, including sex, age, age at onset, family history, psoriatic arthritis (PsA), previous and concomitant immunosuppressant, number of preceding biological treatments and response, time interval between ustekinumab injections and PASI.

### Clinical response to ustekinumab

PASI scores were recorded at baseline; after 4, 16 and 28 weeks of treatment; and at the most recent visit. Responders were defined as 50% reduction in PASI (PASI 50) compared to baseline within 7 months of treatment.

To analyze factors that influence the development of AUA and subsequent clinical response, we defined treatment parameters as follow. “Interrupted therapy” refers to a withdrawal period for more than one month, followed by a retreatment. “Switchers” are patients who switched to ustekinumab after prior treatment with TNF-α blockers (e.g., etanercept or adalimumab).

### Measurement of serum ustekinumab trough concentration and antibodies against ustekinumab and adalimumab

After patients had received at least 7 months (28 weeks) of ustekinumab, blood samples were collected at a single time-point just prior to ustekinumab injection during their routine clinic visits. Trough serum ustekinumab levels were measured by enzyme linked immunosorbent assay (ELISA) similar to one for adalimumab [20] using the target (IL-12) and rabbit antiustekinumab to capture and detect, respectively. AUAs were detected by radioimmunoassay (RIA). RIA and ELISA were both performed at Sanquin Research, Amsterdam, the Netherlands. The detection limit of the assay for serum ustekinumab concentration is approximately 0.002 mg/L and the antibody test was considered positive when the concentration of AUA exceeded 12 arbitrary units (AU)/mL [21]. The details of the methods used to measure AUA and drug concentration are described in S1 File.

For patients who switched from adalimumab to ustekinumab treatment, blood sample for the measurement of anti-adalimumab antibody (AAA) and serum adalimumab concentration was drawn prior to starting ustekinumab—and after completing at least 3 months of preceding adalimumab therapy. Adalimumab trough concentrations and AAA levels were measured by ELISA and RIA respectively (Sanquin Diagnostic Services) as described previously and the mean cut-off value for positive AAA was set at 12 AU/ml [5,10,20,22,23].

### Statistical Analyses

To detect differences between groups, analyses were conducted using the t-test or Mann-Whitney U test, Wilcoxon rank-sum test for continuous variables and the Fisher’s exact or χ 2 test for discrete variables. The Kolmogorov-Smirnov test was applied to test the normal distribution of all continuous variables. Logistic regression analysis was performed to analyze predictors for AUA development. The threshold for significance was set at P<0.05.

## Results

### Patient characteristics and clinical response to ustekinumab

A total of 76 ustekinumab-treated patients with psoriasis vulgaris were enrolled in the study. The demographic data and the baseline characteristics are shown in Table 1. Of the study cohort, 49 (64.5%) and 33(43.4%) of patients achieved at least PASI50 and 75 responses within 7 months of treatment, respectively, similar to previous findings in clinical practice. [16,24]

**Table 1:** Clinical response and associated characteristics of patients with and without anti-ustekinumab antibody.

Cohort characteristics	Patients without AUA (n = 71)	Patients with AUA (n = 5)	P-Value	All patients (n = 76)
**Clinical features**				
Age (years), Mean ± SD	45.7±11.5	53.0± 21.1	0.71	46.2±12.2
Gender (Male/Female)	54/17	3/2	0.6	57/19
Body mass index, Mean ± SD	26.4 ± 4.6	30.0 ± 4.1	0.09	26.7 ± 4.6
Smoking, n (%)	30(42.3%)	3(60%)	0.65	33 (43.4%)
Duration of psoriasis (years), Mean ± SD	16.2±7.6	22.2±8.1	0.14	16.6±7.7
PASI at baseline	16.7±9.9	15.9±11.2	0.70	16.7±9.9
Psoriatic arthritis, n (%)	31(43.7%)	1(20%)	0.39	32 (42.1%)
Erythroderma, n (%)	14(19.7%)	0(0%)	0.58	14 (18.4%)
The number of previous biologics used	0.79±0.86	0.60±0.55	0.79	0.78±0.8
The number of previous systemic traditional antipsoriatic therapy used	2.56±1.24	2.60±1.14	0.95	2.6± 1.2
**Treatment pattern**				
Biologics switching, n (%)	36(50.7%)	3(60%)	>0.99	39 (51.3%)
Concomitant methotrexate, n (%)	21(29.6%)	1(20%)	>0.99	22 (28.9%)
Dose of concomitant methotrexate (mg), Mean ± SD	12.3± 3.5	15±0	0.46	12.4 ±3.6
Duration of biologics treatment (months) prior to AUA measurement, Mean ± SD	13.6±12.2	8.8±2.68	0.37	13.2±11.8
**Treatment response**				
>PASI 50 Responder, n (%)	49(69.0%)	0(0%)	0.004*	49 (64.5%)

AUA, anti-ustekinumab antibody; PASI, psoriasis area and severity index

*P<0.05 (in bold).

### Correlation of anti-ustekinumab antibody levels and their correlation with therapeutic response

After 13.2 ± 11.8 months (mean ± standard deviation (SD); range: 7–61) of ustekinumab treatment, AUA was measured and detected in 5 patients (6.6%). the AUA titers were 41, 1540, 27, 43 and 39 AU/mL, respectively. Of the 5 patients with positive AUA (AUA+), an 88-year-old male with a 15-years history of psoriasis vulgaris had the highest AUA titer (1540 AU/ml) after 13 months treatment. He was diagnosed with bullous pemphigoid 6 years after psoriasis presentation. He responded poorly to methotrexate, acitretin and ultraviolet B phototherapy. After he failed ustekinumab, he switched to adalimumab and golimumab for 6 and 3 months, respectively. He failed them both as well and ultimately died of pneumonia one month later. Comparing AUA+ and patients with negative AUA (AUA-), AUA- patients had higher PASI 50 response but none of AUA+ patients achieved PASI50 or 75 at 7 months (Table 1). Compared to AUA- patients, AUA+ patients had lower mean PASI improvement at week 4 (19.7% vs. 39.7%, p = 0.12), week 16 (16.8% vs. 53.0%, p = 0.02) and week 28 (18.1% vs. 55.7%, p = 0.02).

### The association among ustekinumab trough concentration, AUA levels and clinical response

**Fig 1:**
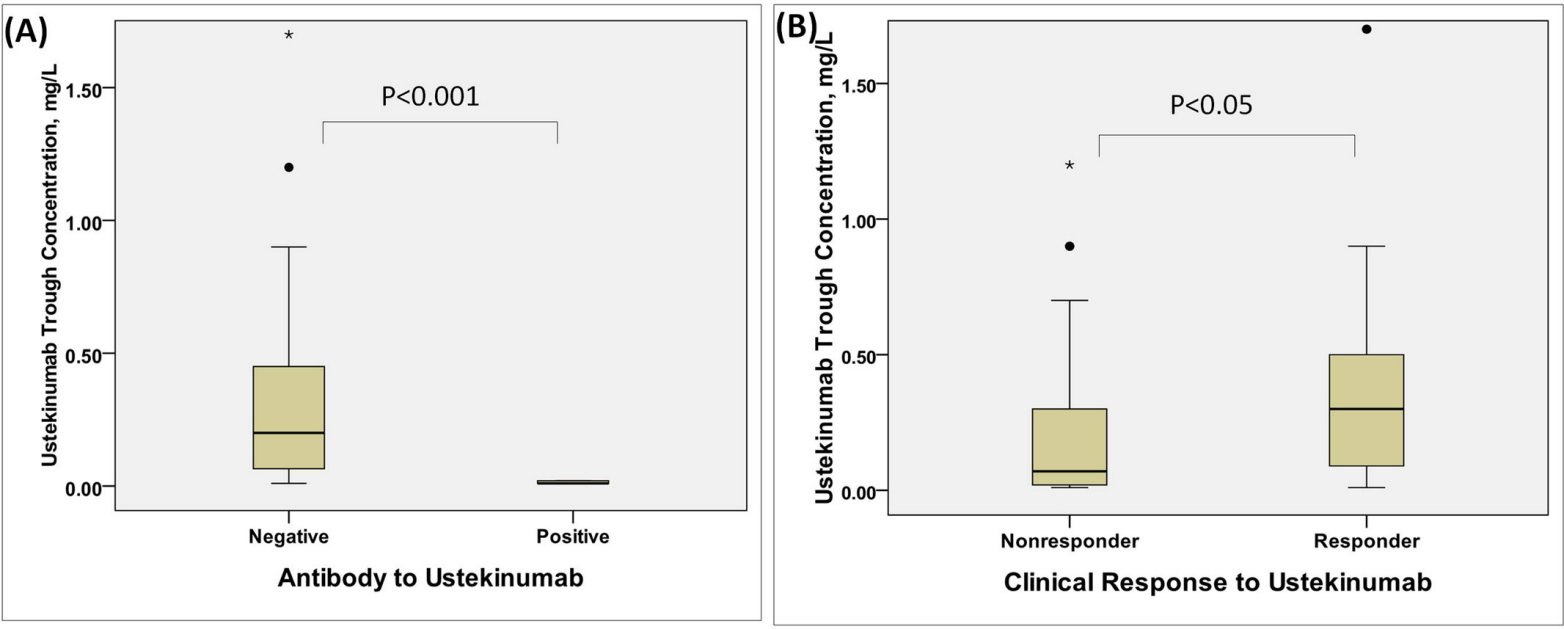
Ustekinumab trough concentration are shown by responder status and anti-ustekinumab antibody (A) Patients with negative anti-ustekinumab antibody had significantly higher drug trough levels compared with those with positive anti-ustekinumab antibody. (B) Nonresponders attained significantly lower ustekinumab trough levels than responders. Responders were defined as 50% reduction in psoriasis area and severity index (PASI 50) compared to baseline within 7 months of treatment.

The median trough level was 0.20 mg/L (interquartile-range (IQR) 0.023–0.4) in all ustekinumab-treated patients. AUA- patients had significantly higher drug trough levels compared with AUA + patients (median = 0.2 mg/L, IQR 0.065–0.5 mg/L vs. median = 0.01 mg/L, IQR 0.01–0.02 mg/L, p<0.001). Responders had significantly higher serum mean ustekinumab concentrations than non-responders (median = 0.3 mg/L, IQR 0.09–0.5 mg/L vs. median = 0.07 mg/L, IQR 0.02–0.3 mg/L, p = 0.03) (Fig 1). In the univariate logistic regression analysis, PASI improvement at week 28 was significantly associated with the AUA development (OR = 0.97; p = 0.035). However, due to small sample of AUA+ patients (resulting in large standard error and wide confidence interval), the influence of drug level, PASI improvement at week 4 and week 16 on the AUA development did not reach statistically significant levels.

### Factors potentially affecting the immunogenicity of ustekinumab

Concomitant methotrexate use was observed in 22 (28.9%) of all ustekinumab-treated patients with a mean (± SD) dose of 12.4 (±3.5) mg weekly (range: 7.5–15). Twenty-one (29.6%) AUA- patients and one (20%) AUA+ patient used methotrexate concurrently with ustekinumab. Therefore, with only one AUA+ patient using methotrexate, the effect of concurrent methotrexate on immunogenicity could not be determined (Table 1). Moreover, there were no statistical difference between AUA+ and AUA- patients in the following: the percentage of male gender, PsA, smoking, PASI at baseline, body mass index, number of previous systemic therapies and duration of ustekinumab treatment. Of the 76 study patients, treatment was interrupted in 18 (23.7%) patients. Loss of health insurance coverage (8/18, 44%) was the most common cause, followed by time constraints (5/18, 28%), financial constraint (3/18, 17%), planning for pregnancy (1/18, 6%) and poor treatment response (1/18, 6%). Treatment pattern in ustekinumab, such as the incidence of treatment interruption and biologics switching, was comparable in patients with and without AUA (Table 1).

### The association between anti-adalimumab antibodies and response after switching to ustekinumab

Of the 39 switchers, 29 patients had switched from adalimumab to ustekinumab. From the adalimumab-to-ustekinumab switchers, 15 patient samples were measured by RIA [5,10,22] to detect AAA prior to ustekinumab initiation. 46.6% (7/15) of the adalimumab-to-ustekinumab switchers were AAA+. The percentage of AUA formation was similar between AAA+ and AAA- patients (14.3% vs. 12.5%, p>0.999). However, compared to AAA+ switchers, AAA- switchers had higher PASI50 (71.4% vs. 37.5%) and PASI75 response (42.9% vs.12.5%) within 7 months of ustekinumab treatment, though difference was not statistically significant (Table 2).

**Table 2:** The immunogenicity and efficacy of ustekinumab in switchers with and without anti-adalimumab antibody.

	Switchers with AAA (n = 7)	Switchers without AAA (n = 8)	p-Value
Antibody to ustekinumab			
AUA (+)	1/7 (14.3%)	1/8 (12.5%)	>0.99
Clinical response to ustekinumab			
>PASI 50 response	3/8 (37.5%)	5/7 (71.4%)	0.32

AAA, anti-adalimumab antibody; AUA, anti-ustekinumab antibody.

## Discussion

Compared to TNF-α blockers, fewer studies have investigate the immunogenicity of ustekinumab, presumably because it is a relatively newer agent and test kits for AUA and drug level are not commercially available. Our study showed that a small proportion (6.5%) of psoriasis on ustekinumab developed AUA in a real clinical setting—similar results have been reported in pivotal studies (3.8% to 6%) [25,26,27,28,29,30,31,32,33]. In addition, the development of AUA was associated with low ustekinumab trough concentrations and impaired treatment outcome.

Many of these studies evaluated the association of AUA and adverse events (e.g., injection site reactions) but not AUA impact on ustekinumab efficacy. Only two studies suggested a possible association between AUA and reduced clinical response [27,29]. Papp et al. showed 5.4% of patients from PHOENIX 2 trial developed AUA after 52 weeks of treatment and most of the AUAs (proportion not specified) were neutralizing. AUA was detected 12.7% (20/158) of PASI 50 responders, compared with 2.0% (12/589) of PASI 75 responders [27]. Tsai et al. reported that 4.4% (5/113) of patients from PEARL trial developed AUA at week 36 and found that a lower proportion of patients (60.0%) achieved PASI 75 in the antibody-positive group (n = 5) compared with 74.5% of patients who were antibody-negative (n = 106) at week 28 [29]. A recent study summarized the immunogenicity results from three Phase 3 randomized controlled trial (RCT trials with ustekinumab in moderate-to-severe psoriasis (PHOENIX 1, PHOENIX 2, and ACCEPT)) [34]. The authors showed that overall incidence of AUA was 5% and the majority (76%) of these AUAs were neutralizing. The titers of AUA were predominantly low (≤1:80) and patients with AUAs tended to have lower serum drug concentrations and a reduced response to ustekinumab [34].

However, patient characteristics in these (RCT) may not accurately reflect actual clinical population and therefore the findings may not be completely applicable in real-world clinical practice. For example, patients with significant co-morbidities are a fact of daily clinical practice, yet they are typically excluded from psoriasis clinical trials.

Psoriasis patients enrolled in RCTs are not accurate representatives of ustekinumab-treated psoriasis patients in routine clinical settings [35]. Moreover, treatment interruption, wide variation in baseline disease severity, switching among biological drugs, and concomitant medication—common in daily practice and presumably affected the risk of AUA development—are excluded in clinical trials. Previous studies suggested antibody positivity did not preclude an efficacy response [29,34]. In contrast, our study showed patients with AUA had a poor response to ustekinumab compare to patients without AUA. This discrepancy may be attributed to different testing methods for AUA, ethnic differences, a real clinical setting and a higher titer of AUA in our study [10,36].

More data have shown that the use of different ADA assays and timing of sample collection in relation to drug administration may contribute to variation in ADA detection [20,22]. Many studies employed ELISA or RIA to measure ADA levels to TNF-α blockers [25,26,27,30,32,33,37]. Two-site (bridging) ELISA is highly specific and sensitive, but it is highly susceptible to interference by other drug in the serum that form immune complexes [37]. In contrast, RIA that we used in this study is a more sensitive than ELISA and less susceptible to drug interference [22]. However, most previous studies are based on ELISA or assays that lacked explanations of methods used. Moreover, the timing of serum sampling with reference to the timing of blood draw and ustekinumab administration was not specified in many of previous reports [28,29,31].

Our study also showed that AAA + switchers had limited response to ustekinumab compared to AAA- switchers, though the difference was not statistically significant, probably due to the small sample size. Unlike AAA+ switchers who failed to maintain the initial response to TNF-α blockers due to antibody formation that accelerated drug clearance, it is possible that TNF is not the main cytokine instigating disease activity in AAA- switchers. Therefore, compared to AAA+ patients, AAA- patients will theoretically have a better response to a drug with a different mode of action (MOA), e.g., ustekinumab. The results of the current study support this hypothesis. However, previous research suggested that patients who previously formed antibodies against first biologics are more likely to develop ADA against the new biopharmaceutical [38]. It was also possible that patients who developed AAAs are more likely to develop AUAs regardless of MOA in the present study. Nevertheless, in this study the rate of AUA formation was similar between switchers with and without AAA. The positive correlation between ADA to different biologics was not found. Thus, we hypothesized that there are different types of non-responders with different underlying mechanisms causing non-response, leading to switch treatment for psoriasis.

Several limitations of our study include the observational cohort design and inadequate statistical power (<50%) from small sample size of patient with AUA. Also each patient’s blood sample was collected once during a clinic visit, though the timing of the collection was not uniform across all samples. Nevertheless, a prior study has shown that most ADA developed before week 24 of treatment [39] and the incidence of ADA remained unchanged over time [34].

Our study explores the clinical utility of AUA detection in daily practice. Since AUA is associated with decrease in both clinical response and ustekinumab concentration, it may be a clinical clue and a potential marker for nonresponse or loss of response. However, the finding in this study was limited by the small sample of AUA+ patients. Additional studies involving a larger sample size, longer follow-up, and serial sampling is needed to strengthen our conclusion. Apropos of switching biologics, the development of AAA from preceding adalimumab therapy did not increase the risk of AUA in subsequent ustekinumab therapy, though it did affect response to ustekinumab. Patients who failed TNF inhibition not due to immunogenic ADA may benefit from switching to an agent of different MOA, e.g., IL-12/23 antagonist—indicating that the status of immunogenicity to previous biological treatment might be associated with the probability of success in subsequent switching. Thus, assessing the immunogenic status in psoriasis patients treated with ustekinumab may be a rational approach for further decision-making and might assist in choosing an optimized treatment modality for the individual patient.

## Supporting Information

S1 File The details of the methods used to measure anti-ustekinumab antibody and drug concentration. (DOC)

## References

[1] Nestle FO, Kaplan DH, Barker J. (2009) Psoriasis. N Engl J Med 361: 496–509. 10.1056/NEJMra0804595 19641206

[2] Mitra A, Fallen RS, Lima HC. (2013) Cytokine-based therapy in psoriasis. Clin Rev Allergy Immunol 44: 173–182. 10.1007/s12016-012-8306-2 22426927

[3] Chiu H-Y, Cheng Y-P, Tsai T-F. (2012) T helper type 17 in psoriasis: From basic immunology to clinical practice. Dermatologica Sinica 30: 136–141.

[4] Sandoval LF, Pierce A, Feldman SR. (2014) Systemic therapies for psoriasis: an evidence-based update. Am J Clin Dermatol 15: 165–180. 10.1007/s40257-014-0064-x 24496885

[5] Bartelds GM, Krieckaert CL, Nurmohamed MT, van Schouwenburg PA, Lems WF, Twisk JW, et al (2011) Development of antidrug antibodies against adalimumab and association with disease activity and treatment failure during long-term follow-up. JAMA 305: 1460–1468. 10.1001/jama.2011.406 21486979

[6] Baert F, Noman M, Vermeire S, Van Assche G, G DH, Carbonez A, et al (2003) Influence of immunogenicity on the long-term efficacy of infliximab in Crohn's disease. N Engl J Med 348: 601–608. 12584368 10.1056/NEJMoa020888

[7] de Vries MK, Wolbink GJ, Stapel SO, de Groot ER, Dijkmans BA, Aarden LA, et al (2007) Inefficacy of infliximab in ankylosing spondylitis is correlated with antibody formation. Ann Rheum Dis 66: 133–134. 17178760 10.1136/ard.2006.057745 PMC1798422

[8] de Vries MK, Brouwer E, van der Horst-Bruinsma IE, Spoorenberg A, van Denderen JC, Jamnitski A, et al (2009) Decreased clinical response to adalimumab in ankylosing spondylitis is associated with antibody formation. Ann Rheum Dis 68: 1787–1788. 10.1136/ard.2009.109702 19822712

[9] Hsu L, Snodgrass BT, Armstrong AW. (2014) Antidrug antibodies in psoriasis: a systematic review. Br J Dermatol 170: 261–273. 10.1111/bjd.12654 24117166

[10] Chiu HY, Wang TS, Chan CC, Lin SJ, Tsai TF. (2015) Risk Factor Analysis for the Immunogenicity of Adalimumab Associated with Decreased Clinical Response in Chinese Patients with Psoriasis. Acta Derm Venereol 95:711–716. 10.2340/00015555-2069 25673333

[11] Papp KA, Griffiths CE, Gordon K, Lebwohl M, Szapary PO, Wasfi Y, et al (2013) Long-term safety of ustekinumab in patients with moderate-to-severe psoriasis: final results from 5 years of follow-up. Br J Dermatol 168: 844–854. 10.1111/bjd.12214 23301632

[12] Tsai TF, Ho V, Song M, Szapary P, Kato T, Wasfi Y, et al (2012) The safety of ustekinumab treatment in patients with moderate-to-severe psoriasis and latent tuberculosis infection. Br J Dermatol 167: 1145–1152. 10.1111/j.1365-2133.2012.11142.x 22803615

[13] Tsai TF, Song M, Shen YK, Schenkel B, Choe YB, Kim NI, et al (2012) Ustekinumab improves health-related quality of life in Korean and Taiwanese patients with moderate to severe psoriasis: results from the PEARL trial. J Drugs Dermatol 11: 943–949. 22859239

[14] Igarashi A, Kato T, Kato M, Song M, Nakagawa H, Japanese Ustekinumab Study Group, et al (2012) Efficacy and safety of ustekinumab in Japanese patients with moderate-to-severe plaque-type psoriasis: long-term results from a phase 2/3 clinical trial. J Dermatol 39: 242–252. 10.1111/j.1346-8138.2011.01347.x 21955098

[15] Chiu HY, Wang TS, Chan CC, Cheng YP, Lin SJ, Tsai TF. (2014) Human leucocyte antigen-Cw6 as a predictor for clinical response to ustekinumab, an interleukin-12/23 blocker, in Chinese patients with psoriasis: a retrospective analysis. Br J Dermatol 171: 1181–1188. 10.1111/bjd.13056 24734995

[16] Wang T-C, Chiu H-Y, Wang T-S, Tsai T-F. (2015) Practical experience of ustekinumab in patients with moderate-to-severe psoriasis who had inadequate therapeutic response to previous tumor necrosis factor blockers. Dermatologica Sinica 33: 5–10.

[17] Chiu HY, Chen CH, Wu MS, Cheng YP, Tsai TF. (2013) The safety profile of ustekinumab in the treatment of patients with psoriasis and concurrent hepatitis B or C. Br J Dermatol 169: 1295–1303. 10.1111/bjd.12461 23746170

[18] Chen DY, Chen YM, Tsai WC, Tseng JC, Chen YH, Hsieh CW, et al (2015) Significant associations of antidrug antibody levels with serum drug trough levels and therapeutic response of adalimumab and etanercept treatment in rheumatoid arthritis. Ann Rheum Dis 74: e16 10.1136/annrheumdis-2013-203893 24442879

[19] Lecluse LL, Driessen RJ, Spuls PI, de Jong EM, Stapel SO, van Doorn MB, et al (2010) Extent and clinical consequences of antibody formation against adalimumab in patients with plaque psoriasis. Arch Dermatol 146: 127–132. 10.1001/archdermatol.2009.347 20157022

[20] van Schouwenburg PA, Bartelds GM, Hart MH, Aarden L, Wolbink GJ, Wouters D, et al (2010) A novel method for the detection of antibodies to adalimumab in the presence of drug reveals "hidden" immunogenicity in rheumatoid arthritis patients. J Immunol Methods 362: 82–88. 10.1016/j.jim.2010.09.005 20833178

[21] Kneepkens EL, Wei JC, Nurmohamed MT, Yeo KJ, Chen CY, van der Horst-Bruinsma IE, et al (2013) Immunogenicity, adalimumab levels and clinical response in ankylosing spondylitis patients during 24 weeks of follow-up. Ann Rheum Dis. 10.1136/annrheumdis-2013-204185 24326011

[22] Hart MH, de Vrieze H, Wouters D, Wolbink GJ, Killestein J, de Groot ER, et al (2011) Differential effect of drug interference in immunogenicity assays. J Immunol Methods 372: 196–203. 10.1016/j.jim.2011.07.019 21824477

[23] Jamnitski A, Krieckaert CL, Nurmohamed MT, Hart MH, Dijkmans BA, Aarden L, et al (2012) Patients non-responding to etanercept obtain lower etanercept concentrations compared with responding patients. Ann Rheum Dis 71: 88–91. 10.1136/annrheumdis-2011-200184 21914626

[24] Ruiz Salas V, Puig L, Alomar A. (2012) Ustekinumab in clinical practice: response depends on dose and previous treatment. J Eur Acad Dermatol Venereol 26: 508–513. 10.1111/j.1468-3083.2011.04325.x 22077903

[25] Kauffman CL, Aria N, Toichi E, McCormick TS, Cooper KD, Gottlieb AB, et al (2004) A phase I study evaluating the safety, pharmacokinetics, and clinical response of a human IL-12 p40 antibody in subjects with plaque psoriasis. J Invest Dermatol 123: 1037–1044. 15610511 10.1111/j.0022-202X.2004.23448.x

[26] Krueger GG, Langley RG, Leonardi C, Yeilding N, Guzzo C, Wang Y, et al (2007) A human interleukin-12/23 monoclonal antibody for the treatment of psoriasis. N Engl J Med 356: 580–592. 17287478 10.1056/NEJMoa062382

[27] Papp KA, Langley RG, Lebwohl M, Krueger GG, Szapary P, Yeilding N, et al (2008) Efficacy and safety of ustekinumab, a human interleukin-12/23 monoclonal antibody, in patients with psoriasis: 52-week results from a randomised, double-blind, placebo-controlled trial (PHOENIX 2). Lancet 371: 1675–1684. 10.1016/S0140-6736(08)60726-6 18486740

[28] Griffiths CE, Strober BE, van de Kerkhof P, Ho V, Fidelus-Gort R, Yeilding N, et al (2010) Comparison of ustekinumab and etanercept for moderate-to-severe psoriasis. N Engl J Med 362: 118–128. 10.1056/NEJMoa0810652 20071701

[29] Tsai TF, Ho JC, Song M, Szapary P, Guzzo C, Shen YK, et al (2011) Efficacy and safety of ustekinumab for the treatment of moderate-to-severe psoriasis: a phase III, randomized, placebo-controlled trial in Taiwanese and Korean patients (PEARL). J Dermatol Sci 63: 154–163. 10.1016/j.jdermsci.2011.05.005 21741220

[30] Kimball AB, Papp KA, Wasfi Y, Chan D, Bissonnette R, Sofen H, et al (2013) Long-term efficacy of ustekinumab in patients with moderate-to-severe psoriasis treated for up to 5 years in the PHOENIX 1 study. J Eur Acad Dermatol Venereol 27: 1535–1545. 10.1111/jdv.12046 23279003

[31] Zhu X, Zheng M, Song M, Shen YK, Chan D, Szapary PO, et al (2013) Efficacy and safety of ustekinumab in Chinese patients with moderate to severe plaque-type psoriasis: results from a phase 3 clinical trial (LOTUS). J Drugs Dermatol 12: 166–174. 23377389

[32] Leonardi CL, Kimball AB, Papp KA, Yeilding N, Guzzo C, Wang Y, et al (2008) Efficacy and safety of ustekinumab, a human interleukin-12/23 monoclonal antibody, in patients with psoriasis: 76-week results from a randomised, double-blind, placebo-controlled trial (PHOENIX 1). Lancet 371: 1665–1674. 10.1016/S0140-6736(08)60725-4 18486739

[33] Langley RG, Lebwohl M, Krueger GG, Szapary PO, Wasfi Y, Chan D, et al (2014) Long-term efficacy and safety of ustekinumab, with and without dosing adjustment, in patients with moderate-to-severe psoriasis: Results from the PHOENIX 2 study through 5 years of follow-up. Br J Dermatol 172:1371–1383. 10.1111/bjd.13469 25307931

[34] Jullien D, Prinz JC, Nestle FO. (2015) Immunogenicity of biotherapy used in psoriasis: the science behind the scenes. J Invest Dermatol 135: 31–38. 10.1038/jid.2014.295 25120005

[35] Chiu HY, Wang TS, Chang CY, Tsai TF. (2012) The effectiveness and safety of adalimumab in the treatment of non-reimbursed patients with mild-to-moderate psoriasis. J Eur Acad Dermatol Venereol 26: 991–998. 10.1111/j.1468-3083.2011.04199.x 21812835

[36] Chiu HY, Huang PY, Jee SH, Hu CY, Chou CT, Chang YT, et al (2012) HLA polymorphism among Chinese patients with chronic plaque psoriasis: subgroup analysis. Br J Dermatol 166: 288–297. 10.1111/j.1365-2133.2011.10688.x 21985130

[37] Wolbink GJ, Aarden LA, Dijkmans BA. (2009) Dealing with immunogenicity of biologicals: assessment and clinical relevance. Curr Opin Rheumatol 21: 211–215. 19399992 10.1097/bor.0b013e328329ed8b

[38] Bartelds GM, Wijbrandts CA, Nurmohamed MT, Stapel S, Lems WF, Aarden L, et al (2010) Anti-infliximab and anti-adalimumab antibodies in relation to response to adalimumab in infliximab switchers and anti-tumour necrosis factor naive patients: a cohort study. Ann Rheum Dis 69: 817–821. 10.1136/ard.2009.112847 19581278

[39] Menting SP, van Lumig PP, de Vries AC, van den Reek JM, van der Kleij D, de Jong EM, et al (2014) Extent and consequences of antibody formation against adalimumab in patients with psoriasis: one-year follow-up. JAMA Dermatol 150: 130–136. 10.1001/jamadermatol.2013.8347 24352354

