# Supplementary material for: The Association between Clinical Response to Ustekinumab and Immunogenicity to Ustekinumab and Prior Adalimumab
Source: PLoS One. 2015 Nov 13;10(11):e0142930. doi: 10.1371/journal.pone.0142930 (PMC4643875; doi:10.1371/journal.pone.0142930)
Supplement: S1 File — (DOC) [file pone.0142930.s001.doc]

**Measurement of ustekinumab levels**

To measure ustekinumab levels an immunoassay was set up analogously to an assay for measuring adalimumab (1), using the target (IL-12) to capture ustekinumab, and rabbit anti-ustekinumab for detection. Maxisorp ELISA plates were coated overnight at room temperature with 0.5 μg/ml mouse anti-IL-12 (Diaclone) in PBS. Plates were washed five times with PBS/ 0.02% Tween (PT), followed by incubation for 1 h at RT with recombinant human IL-12 (10 ng/ml) (R&D systems) diluted in high performance ELISA buffer (HPE, Sanquin). Next, the plates were washed and incubated for 1 h with patient serum which was serially diluted in HPE. After washing 5 times with PT, plates were incubated for 1 h with biotinylated ustekinumab specific rabbit anti-idiotype (25 ng/ml in HPE). The rabbit anti-ustekinumab antibodies were produced analogously as described for natalizumab (2). After washing, streptavidin-poly-HRP (Sanquin) (1:10,000, in HPE) was added for 1 h at 37 °C. After washing the ELISA was developed with 100 μg/ml tetramethylbenzidine in 0.11 M sodium acetate (pH 5.5) containing 0.003% (v/v) H_2_O_2_. The reaction was stopped with 2 M H_2_SO_4_. Absorption was measured at 450 nm related to a titration curve of ustekinumab in each plate.

**Measurement of anti-ustekinumab antibodies**

Measurement of anti-ustekinumab antibodies was essentially carried out as described before (3,4). One microliter of serum diluted in Freeze buffer (Sanquin) (4) was incubated overnight with 1 mg Protein A Sepharose (GE healthcare, Chalfont St. Giles, UK) and 2.5 ng biotinylated F(ab')2 ustekinumab in a final volume of 800 μl. Subsequently the samples were washed with PBS 0.005%Tween and ca. 1 ng ^125^I-labeled streptavidin was added in 800 ul final volume of PBS-AT (PBS / 0.01M EDTA / 0.3% Bovine Serum Albumin / 0.004% Tween-20 / 0.05% NaN_3_) and incubated overnight. Unbound label was removed by washing, and Sepharose-bound radioactivity was measured. Antibody levels were compared to a standard serum containing ADA and expressed in arbitrary units (AU). The assay cut-off of 12 AU/ml was determined based on mean + 3 SD measured in a panel of 50 sera from healthy donors and 15 sera containing anti-CCP, ANA, and/or RF.

**References**

(1) A novel method for the detection of antibodies to adalimumab in the presence of drug reveals "hidden" immunogenicity in rheumatoid arthritis patients. van Schouwenburg PA, Bartelds GM, Hart MH, Aarden L, Wolbink GJ, Wouters D. J Immunol Methods. 2010 Oct 31;362(1-2):82-8.

(2) Measurement of serum levels of natalizumab, an immunoglobulin G4 therapeutic monoclonal antibody. Rispens T, Leeuwen Av, Vennegoor A, Killestein J, Aalberse RC, Wolbink GJ, Aarden LA. Anal Biochem. 2011 Apr 15;411(2):271-6.

(3)Wolbink, G.J., Vis, M., Lems, W., Voskuyl, A.E., de G., E., Nurmohamed, M.T., Stapel, S., Tak, P.P., Aarden, L., Dijkmans, B., 2006. Development of antiinfliximab antibodies and relationship to clinical response in patients with rheumatoid arthritis. Arthritis Rheum. 54, 711.

(4)T. Rispens, V.H. de, G.E. de, D. Wouters, S. Stapel, G.J. Wolbink, L.A. Aarden, Antibodies to constant domains of therapeutic monoclonal antibodies: anti-hinge antibodies in immunogenicity testing. J.Immunol.Methods 375 (2012) 93-99.
